# Heterogeneous impacts of road characteristics on the severity of taxi-involved crashes: Practical insights for safety improvement

**DOI:** 10.1371/journal.pone.0361581

**Published:** 2026-10-28

**Authors:** Jingcai Yu, Peng Lei, Jiacheng Yin, Li Tang, Shunchao Wang

**Affiliations:** 1 School of Automobile and Transportation, Xihua University, Chengdu, Sichuan, China; 2 School of Automotive Engineering, Harbin Institute of Technology, Weihai, Shandong, China; Tsinghua University, CHINA

## Abstract

Taxi traffic safety arises from the complex interplay among road infrastructure, driver behavior, vehicle characteristics, and environmental conditions. However, driver, vehicle, and environmental factors are typically challenging to regulate directly through policy or design, whereas road characteristics can be systematically adjusted through infrastructure planning and traffic management strategies. Therefore, understanding how specific road features contribute to crash risk offers a more practical and sustainable pathway for enhancing taxi travel safety. This study aims at investigating how road characteristics heterogeneously influence the severity of taxi-involved crashes. To accomplish this, a latent class clustering method is first leveraged to classify crashes with similar contextual features. Subsequently, a partial proportional odds model is applied to quantify the class-specific impacts of road characteristics on taxi-involved crash severity. The taxi crash data reported by the London police from January 2019 to June 2024 are utilized to achieve this study. The results demonstrate substantial heterogeneity in how road characteristics affect taxi-involved crash outcomes across different crash classes. Among the examined factors on taxi-involved crashes, speed limits and junction controls exert the most pronounced influences. Higher speed limits (≥40 mph) substantially elevate the probabilities of serious injuries (+14.76%) and fatalities (+0.93%). In contrast, moderate speed limits (31−40 mph) are associated with notably lower marginal effects on serious injuries (+8.27%) and fatalities (+0.46%). The installation of traffic signals at junctions decreases the likelihood of serious injuries (−11.68%) and fatalities (−0.50%). These findings indicate that uniform safety policies may be insufficient for mitigating class-specific taxi risks. This study contributes empirical evidence and practical insights for road safety management. By identifying the heterogeneous influence patterns of road characteristics, the findings support targeted infrastructure design, speed management, and signal control strategies to effectively reduce both the frequency and severity of taxi-related crashes.

## 1 Introduction

Taxis, as an integral component of the road transportation system, play a crucial role in the provision of convenient, fast, and efficient door-to-door services. However, there have been growing concerns about the safety of taxis, as evidenced by a plethora of reports [1–4]. Taking Korean as an example, official data from 2016 to 2020 demonstrate that taxi-involved crashes accounted for approximately 46% of all traffic crashes [5]. Furthermore, compared to 2017, the number of taxi-involved crashes and injuries surged by 1.46% and 0.03% in 2018, respectively. Similar trends have also been observed in other countries, including Ethiopia, Brazil, Iran, and the United States, where taxi-involved transportation faces a heightened risk of crashes, contributing significantly to road traffic accidents [4,6]. Allowing for these circumstances, it is considered crucial to enhance taxi transportation safety by developing targeted countermeasures.

Previous studies on taxi-involved crashes have examined a wide range of contributing factors, including driver, vehicle, environmental, and road characteristics [2,7–9]. These studies have provided valuable insights into the determinants of taxi crash occurrence and severity. However, two issues remain insufficiently addressed. First, road characteristics deserve particular attention because, unlike many driver and environmental factors, they can be directly modified through infrastructure planning, traffic management, and road safety interventions. For example, speed limits, junction control, pedestrian facilities, and road design can be systematically adjusted by transportation authorities. Therefore, identifying the specific associations between road characteristics and taxi-involved crash severity can provide more actionable information for safety interventions. Second, taxi-involved crashes are unlikely to constitute a homogeneous population. Crashes occurring under different combinations of driver, environmental, and vehicle conditions may represent distinct crash contexts, and the same road characteristic may therefore have different effects across these contexts [7–16]. Moreover, crash severity is ordinal in nature, ranging from slight injury to serious and fatal injury. Assuming that an explanatory variable has an identical effect across all severity thresholds may consequently mask potentially important severity-specific relationships [17–21]. These considerations suggest that two forms of heterogeneity should be distinguished: heterogeneity across different crash contexts and heterogeneity across different severity levels within a crash context. Accordingly, an analytical framework that first identifies relatively homogeneous crash contexts and subsequently allows road characteristics to have different effects across severity levels may provide a more informative understanding of taxi-involved crash severity.

The partial proportional odds (PPO) model has been proven to be an appropriate approach in capturing heterogeneous effects of determinants through relaxing the parallel lines (PL) assumption [20,22]. The PPO model is capable of relaxing the parallel lines (PL) assumption. Using this framework, the varying impacts of explanatory variables on taxi-involved crash severity were systematically examined. This approach enables a more comprehensive interpretation of how different factors are associated with distinct levels of crash outcomes.

This study endeavors to reveal the heterogeneous effects of road characteristics on different taxi-involved crash severities. To address this issue, the latent class clustering (LCC) is exploited to divide taxi-involved crash data into a sequence of classes, aiming at minimizing the heterogeneity within each class and maximizing the heterogeneity with classes. Then, the partial proportional odds (PPO) model is leveraged to unravel the heterogeneity roles of road characteristics on the taxi-involved crash severities. Finally, marginal effects are calculated to interpret the heterogeneity effects, and valuable insights are provided to enhance road safety for the taxi industry. The analysis is conducted by applying the taxi-involved crash data collected in London from January 2019 to June 2024.

This study makes two main contributions. First, it identifies heterogeneous crash contexts among taxi-involved crashes by clustering driver, environmental, and vehicle characteristics. Rather than treating all taxi-involved crashes as a homogeneous population, the analysis provides a data-driven characterization of distinct crash profiles and establishes a basis for context-specific safety analysis. Second, the study examines whether the associations between road characteristics and crash severity vary both across crash contexts and across severity levels. By combining latent class clustering with the partial proportional odds model, the analysis provides a framework for distinguishing context-specific and severity-specific effects of road characteristics. This perspective can help transportation practitioners develop more targeted interventions instead of relying solely on uniform safety measures.

The remainder of this study is organized as follows. In Section 2, a review is conducted with respect to taxi-involved crash studies and related methods for crash severity. Section 3 elaborates on taxi-involved crash data. Section 4 outlines the methodology adopted in this study, which consists of two main components: the LCC and the PPO model. In Section 5, the heterogeneous effects of road characteristics are presented and discussed. Section 6 summarizes the findings of this study, with directions for future work indicated.

## 2 Literature review

At present, there are plenty of studies conducted to explore the factors influencing taxi-involved crash severities, as detailed in Table 1. In these studies, a wide range of factors have been examined, including driver characteristics (e.g., gender, age, education), environmental factors (e.g., day, weather, time), and vehicle characteristics (e.g., taxi color, number of vehicles involved) [1,2,4,10,13,19,23]. Notably, a vast majority of these studies have investigated the cross-effects of multiple factors on taxi-involved crash severities. For example, some studies focus on the combined effects of environmental and road factors [24], while others focus on the interactions between driver and environmental factors [4,5,15]. These studies provide valuable insights into the factors contributing to taxi-involved crashes.

**Table 1:** Summary of the contributing factors and methods used in taxi-involved crashes.

References	Contributing factors	Method
Tay and Choi [29]	Driver factors	Binary logistic model
Wu et al. [30]	Driver factors	Binary logistic model
Meng et al. [24]	Environment, vehicle, driver, and road factors	Bayesian space-time logistic model
Wang et al. [31]	Driver and environmental factors	Data statistics and analysis
Husain et al. [32]	Driver factors	Hierarchical linear modeling
Li et al. [10]	Driver and vehicle factors	Descriptive statistics analysis
Ma et al. [7]	Environment factors	Poisson conditional autoregressive models
Mehdizadeh et al. [33]	Driver factors	Principal component analysis
Wang et al. [34]	Driver factors	Hybrid bivariate model
Noh et al. [8]	Driver and vehicle factors	Binary logit model
Peng et al. [35]	Driver factor and financial burden	Zero-inflated Poisson model
Park et al. [5]	Driver and environmental factors	Random forest method
Kang et al. [1]	Environmental factors, vehicle factors, driver factors	Random forest method
Mehri et al. [2]	Driver factors	Grounded theory approach
Peng et al. [15]	Driver and environmental factors	Bayesian network model
Dai et al. [36]	Driver factors	Binomial distribution model
Hussain et al. [23]	Driver factors	Structural equation model
Jaydarifard et al. [19]	Driver factors	Joanna Briggs appraisal
Peng et al. [37]	Driver factors	XGBoost and SHAP
Tamakloe et al. [4]	Driver and environmental factors	Random parameters binary logit framework

Although road characteristics have been considered in several previous studies, their effects have generally been examined together with driver, environmental, and vehicle factors [3,4,8,11,25]. Less attention has been given to how road characteristics operate under different latent crash contexts and whether their effects vary across different levels of crash severity. It has been highlighted in recent studies that considering the heterogeneity of traffic crash data is essential for the analysis of the contributing factors [11,26]. As observed, contradictory conclusions can be reached by analyzing crash data as a whole dataset. For instance, it may be found in one study that male drivers increase the risk of crashes, while the opposite effect may be found in another study. These discrepancies are attributable to the latent and unobserved features of crash data remaining hidden during collective analysis [27,28].

Overall, previous taxi crash studies have made substantial progress in identifying factors associated with crash severity. However, existing approaches differ in their ability to account for heterogeneity. Conventional regression-based models generally estimate average effects across the entire sample, whereas data-driven approaches such as random forests and XGBoost can capture complex relationships but provide less direct interpretation of ordinal severity transitions. Similarly, models that account for unobserved heterogeneity do not necessarily distinguish whether the effects of road characteristics vary across severity levels.

To address this issue, it is crucial to use the techniques applicable to capture the underlying heterogeneity within the data. Currently, latent class clustering (LCC) has been accepted as an effective solution to maximizing the heterogeneity of traffic crash data [20,38]. Through LCC, taxi-involved crash data can be divided into different classes, which facilitates a more accurate and meaningful analysis of the contributing factors. Therefore, LCC is performed in this study to cluster taxi-involved crash data into heterogeneous classes, which is conducive to comprehensively exploring the diverse characteristics and factors associated with different classes.

A range of analytical approaches has been widely adopted in previous studies to investigate taxi-involved crash severity, including binary logistic regression, structural equation modeling, random forest, Bayesian network models, XGBoost, and SHAP techniques [1,4,8,15,37]. Although these methods have demonstrated strong predictive and explanatory capabilities, many of them are developed under the parallel lines (PL) assumption, implying that explanatory variables exert stable and identical effects across different crash severity levels. Nevertheless, such an assumption may be overly restrictive for traffic crash data, which are inherently ordinal and characterized by substantial heterogeneity, particularly when crash outcomes span from minor injuries to fatal crashes.

To overcome the above limitation, the partial proportional odds (PPO) model is applied in this study. Allowing for the relaxation of the parallel line (PL) assumption, the PPO model is capable of capturing the ordinal nature of traffic crash data, with consideration given to the varying effects of contributing factors on different crash severities [20,26]. Through the PPO model, the heterogeneous effects of explanatory variables on taxi-involved crash severity can be examined by considering the ordinal nature of the data. By adopting this approach, a more nuanced understanding is gained in respect of how different factors contribute to the varying levels of crash severity.

In summary, the LCC technique is first applied to cluster taxi-involved crash data into homogeneous classes, which elicits the identification between classes. This improves the understanding of distinct characteristics and patterns within each class. Furthermore, the PPO model is applied to investigate the heterogeneous effects of road characteristics on taxi-involved crash severity within each class. Taking into account the ordinal nature of crash severity, this modeling approach relaxes the PL assumption, which allows for the varying effects of explanatory variables across different crash severities.

## 3 Data

The taxi-involved crash data used in this study were sourced from the London police, spanning the period from January 2019 to June 2024. The data carry information on various categories of the variables related to the taxi-involved crashes, including four aspects: driver characteristics, environmental factors, road characteristics, and vehicle characteristics. Driver characteristics involve gender, age, and travel purpose. Environmental factors include day, time, and weather conditions. Road characteristics incorporate road class, road type, speed limit, junction details, junction control, pedestrian crossing facility, light conditions, road surface, and restricted lane. Vehicle characteristics encompass vehicle maneuver, collision type, and the number of vehicles involved in the accident.

The selection of these variables was aligned with the prior studies conducted to examine taxi-involved crashes and their contributing factors. Furthermore, the selection was influenced by the accessibility and completeness of available data. The taxi-involved crash data provide the details on each severity level, including definitions, descriptions, and the distribution of variables within each severity category. Allowing for a thorough investigation into the effects of these variables on taxi-involved crash severity, this comprehensive dataset lays a solid foundation for the analysis conducted in this study. All variables included in the LCC stage were represented as categorical variables. Where an original variable was continuous, it was categorized into substantively meaningful intervals before clustering. The categorization was based on the categories available in the original crash database and/or established classifications used in previous studies. This preprocessing was conducted to ensure that the latent classes represented interpretable crash contexts.

The original dataset contained 29,541 taxi-involved crash records. Records with missing, unknown, or inconsistent information in the variables required for the subsequent analysis were excluded according to predefined data-cleaning criteria. After this procedure, 9,788 complete records were retained for analysis. The data-cleaning procedure was applied consistently across the variables used in the LCC and PPO analyses. Table 2 presents the descriptive statistics of taxi-involved crash severity. The taxi-involved crash severity was categorized into three levels: fatal injury, serious injury (including evident injury or disabling injury), and slight injury (including no injury or possible injury). The distribution of these severity levels was detailed as follows: fatal injury accounts for 19 cases (0.19%), serious injury accounts for 1,144 cases (11.69%), and slight injury accounts for 8,625 cases (88.12%).

**Table 2:** Descriptive statistics of taxi-involved crash severity.

Variable		Total	Slight	Serious	Fatal
*Taxi-involved crashes*		9788	8625(88.12%)	1144(11.69%)	19(0.19%)
*Driver characteristics*					
Gender	Male	6445	5644(87.57%)	784(12.16%)	17(0.27%)
	Female	3343	2981(89.17%)	360(10.77%)	2(0.06%)
Age	24-	158	135(85.44%)	22(13.92%)	1(0.64%)
	25-34	1222	1085(88.79%)	135(11.05%)	2(0.16%)
	35-64	6293	5506(87.49%)	772(12.27%)	15(0.24%)
	65+	2115	1899(89.79%)	215(10.17%)	1(0.04%)
Travel purpose	Commuting	5484	4811(87.73%)	661(12.05%)	12(0.22%)
	Not commuting	4304	3814(88.62%)	483(11.22%)	7(0.16%)
*Environment characteristics*					
Day	Weekend	2927	2600(88.83%)	321(10.97%)	6(0.20%)
	Weekday	6861	6025(87.82%)	823(12.00%)	13(0.18%)
Time	00:00 - 06:59	1613	1402(86.92%)	205(12.71%)	6(0.37%)
	07:00 - 08:59	763	656(85.98%)	107(14.02%)	0(0.00%)
	09:00 - 11:59	1263	1127(89.23%)	135(10.69%)	1(0.08%)
	12:00 - 16:59	2257	2007(88.92%)	248(10.99%)	2(0.09%)
	17:00 - 19:59	1398	1232(88.13%)	166(11.87%)	0(0.00%)
	20:00 - 23:59	2494	2201(88.25%)	283(11.35%)	10(0.40%)
Weather	Fine	8177	7168(87.66%)	992(12.13%)	17(0.21%)
	Adverse(rain/snow/fog/mist)	1611	1457(90.44%)	152(9.44%)	2(0.12%)
*Road Characteristics*					
Road class	Arterial road and motorway	6105	5360(87.80%)	732(11.99%)	13(0.21%)
	Sub-arterial road	814	718(88.21%)	94(11.54%)	2(0.25%)
	Side way	2869	2547(88.78%)	318(11.08%)	4(0.14%)
Road type	Roundabout	464	431(92.89%)	33(7.11%)	0(0.00%)
	One-way street	1924	1758(91.37%)	165(8.58%)	1(0.05%)
	Single carriageway	5249	4522(86.15%)	714(13.60%)	13(0.25%)
	Dual carriageway	2151	1914(88.98%)	232(10.78%)	5(0.22%)
Speed limit	≤20 mph	3608	3178(88.08%)	425(11.78%)	5(0.14%)
	21-30 mph	5643	4965(87.99%)	667(11.82%)	11(0.19%)
	31-40 mph	355	319(89.86%)	35(9.86%)	1(0.28%)
	≥40 mph	182	163(89.56%)	17(9.34%)	2(1.10%)
Junction details	No junction in 20m	2139	1864(87.14%)	269(12.58%)	6(0.28%)
	Roundabout	598	554(92.64%)	43(7.19%)	1(0.17%)
	T/staggered junction	3732	3243(86.90%)	484(12.97%)	5(0.13%)
	Crossroads	1475	1314(89.09%)	158(10.71%)	3(0.20%)
	More than 4 arms	1844	1650(89.48%)	190(10.30%)	4(0.22%)
Junction Control	Not applicable	1414	1240(87.69%)	169(11.95%)	5(0.36%)
	Traffic signal	3442	3105(90.21%)	332(9.65%)	5(0.15%)
	Stop sign and give way	4932	4280(86.78%)	643(13.04%)	9(0.18%)
Pedestrian crossing	No in 50 meters	5518	4920(89.16%)	587(10.64%)	11(0.20%)
	Zebra/pelican/central refuge	2071	1774(85.66%)	293(14.15%)	4(0.19%)
	Pedestrian phase with signal	2199	1931(87.81%)	264(12.01%)	4(0.18%)
Light conditions	Daylight	5646	5004(88.63%)	637(11.28%)	5(0.09%)
	Dark	4142	3621(87.42%)	507(12.24%)	14(0.34%)
Road surface	Dry	7389	6459(87.41%)	919(12.44%)	11(0.15%)
	Wet	2043	1824(89.28%)	212(10.38%)	7(0.34%)
	Ice/snow	356	342(96.07%)	13(3.65%)	1(0.28%)
Restricted lane	Unrestricted lane	7356	6357(86.42%)	982(13.35%)	17(0.23%)
	Motorized vehicle lanes	1681	1589(94.53%)	91(5.42%)	1(0.05%)
	Non-motorized vehicle lanes	751	679(90.41%)	71(9.47%)	1(0.12%)
*Vehicle characteristics*					
Vehicle maneuver	Going ahead	3410	3004(88.09%)	401(11.76%)	5(0.15%)
	Turning and changing lane	3010	2619(87.01%)	382(12.69%)	9(0.30%)
	Slowing or stopping	3368	3002(89.13%)	361(10.72%)	5(0.15%)
Collision type	Did not impact	451	398(88.25%)	53(11.75%)	0(0.00%)
	Rear-end	5696	4997(87.73%)	687(12.06%)	12(0.21%)
	Near/Off-side	3641	3230(88.71%)	404(11.10%)	7(0.19%)
Vehicles in an accident	Single vehicle	1993	1645(82.54%)	342(17.16%)	6(0.30%)
	Multiple vehicles	7795	6980(89.54%)	802(10.29%)	13(0.17%)

According to the statistics showcased in Table 2, there are several variables associated with taxi-involved severe injuries. Age is a crucial determinant in taxi-involved crash severity. By comparing different age groups, it can be discovered that drivers aged 24 and under account for a higher percentage of fatal injuries (0.64%) compared to other age groups. Moreover, the speed limit is found to be another critical variable associated with taxi-involved crash severity. As displayed in Table 2, the percentage of fatal injuries increases with higher speed limits. For instance, there is a larger percentage of fatal injuries for speed limits over 40 mph (1.10%) than for speed limits below 20 mph (0.14%). Furthermore, junction control exhibits a significant correlation with taxi-involved crash severity, with the percentage of fatal injuries at junctions without control reaching 0.36%. As unraveled in Table 2, adverse road surfaces and dark light conditions are the major contributing factors to taxi-involved crash severity.

## 4 Methodology

### 4.1 Latent class clustering

The LCC technique is a statistical approach that partitions multivariate categorical data into distinct groups, maximizing within-group similarity and between-group heterogeneity [39]. A key advantage of this approach is its ability to handle diverse data types—categorical, nominal, count, and continuous—without the need for variable standardization [40,41]. By applying LCC to the taxi-involved crash data, we can identify unobserved patterns or features within the data and group similar cases together based on their characteristics.

In this study, continuous variables for taxi-involved crash data are considered discrete variables for characterizing the scenarios. Based on the crash data, *M* latent classes are assumed to be evaluated in *n* category variables.


$$\begin{array}{c} P\left(Y_{i}=y\right)=\sum_{m=\text{1}}^{M}\theta_{m}\prod_{n=\text{1}}^{N}\prod_{r_{n}=\text{1}}^{R_{n}}\alpha_{n,r_{n}|m}^{S({y_{n}=r}_{n})} \end{array}$$ (1)

where $\theta_{m}$ represents the probability in Class *m* (*m* = 1, 2, …, *M*). Crash *i* involves *N* (*N* = 9) category variables, including driver characteristics (age, gender, and travel purpose), environment characteristics (day, time, and weather), and vehicle characteristics (vehicle maneuver, collision type, and the number of vehicles in an accident). $Y_{i}$ **(**$Y_{i}$=1, 2, …$\;r_{n}$**)** is the result of a crash *i* for the *N* category variable. $\alpha_{n,r_{n}|m}^{S({y_{n}=r}_{n})}$ denotes the response probability that crash *i* has the attribute $r_{n}$, conditioned on Class *m*. $S({y_{n}=r}_{n})$ indicates the indicator function that equals 1 when ${y_{n}=r}_{n}$, and 0 otherwise [42,43]. $\theta_{m}$ denotes the multinomial logit model, which is expressed as:


$$\begin{array}{c} \theta_{m}=P(m)=\frac{e^{\omega_{om}+\omega_{\text{1}m}x_{i}}}{\text{1}+\sum_{\mu =\text{1}}^{M-\text{1}}e^{\omega_{o\mu }+\omega_{\text{1}\mu }x_{i}}} \end{array}$$ (2)

where $x_{i}$ denotes the attribute vector of crash *i*, ω represents the coefficient vector. µ denotes the number of parameters to be estimated.

This study harnesses Entropy value and three criteria to identify the number of classes, including Akaike Information Criterion (AIC), Bayesian Information Criterion (BIC), and sample-sized adjusted BIC (ABIC) [39,43].


$$\begin{array}{c} AIC=-\text{2}log\left(\frac{SSE}{m}\right)+\text{2}\mu \end{array}$$ (3)

$$\begin{array}{c} BIC=-\text{2}log\left(\frac{SSE}{m}\right)+log(mmu \end{array}$$ (4)

$$\begin{array}{c} ABIC=-\text{2}log\left(\frac{SSE}{m}\right)+\mu \cdot log\left((m)+\text{1}\right) \end{array}$$ (5)

$$\begin{array}{c} Entropy=-\sum_{i=\text{1}}^{m}p_{i}\text{ln}(p_{i}) \end{array}$$ (6)

where SSE denotes the error sum of squares. µ is the number of parameters to be evaluated. $p_{i}$ represents the probability of a variable $x_{i}$.

The four indicators are simultaneously utilized to derive the classification outcomes. The entropy value surpasses the recommended benchmark of 0.9, while the percentage reductions in the three evaluation criteria remain below 1%, demonstrating the stability and reliability of the classification results. The Entropy value is a measure of the data quality obtained from the LCC process in clustering the taxi crash data. It ranges from 0 to 1, with a value close to 1 indicating a better clustering result [42,43]. In addition, three criteria (i.e., BIC, AIC, and ABIC) are used to uncover the quality of the clustering model. These criteria represent the error between the evaluated likelihood function and the true likelihood function, and smaller values indicate a better fit. However, it should be noted that for large datasets such as traffic data, the minimum values of these criteria may not always be achieved as the number of classes increases [26]. Hence, in determining the appropriate number of classes, it is recommended to consider both the Entropy value and the percentage reduction (less than 1%) in the three criteria.

### 4.2 Partial proportional odds model

Indeed, the ordered logit model, which assumes the PL assumption, assumes that the effects of variables on the outcome are constant and the same across all levels of the outcome variable [19,20]. However, this assumption may not hold in many real-world scenarios where the effects of variables vary across different levels or categories of the outcome variable [44]. In the case of taxi-involved crash severity, the severity levels (i.e., slight, serious, and fatal injury) have a natural ordering, and the effects of explanatory variables likely differ across these severity levels.

To address this issue and capture the ordinal nature of the crash severity, several models (e.g., Random-parameter model, Bayesian model, and partial proportional odds model) have been proposed. Random-parameter models and Bayesian models are more flexible than the PPO model. However, they are more complex due to the inclusion of a greater number of parameters and model structures. Especially, prior knowledge is needed for Bayesian models, which is challenging. The PPO model has been frequently employed in identifying the heterogeneities of traffic crashes (e.g.[19,20,45],). For instance, Awasthi et al. [20] applied the PPO model to address how the spatial relationships affect the severity of curve-induced crashes.

This study aims at addressing the heterogeneous effects of road characteristics on taxi-involved crash severity. The PPO model is an extension of the ordered logit model that relaxes the PL assumption and allows for the effects of variables to vary across different levels of the outcome variable. By estimating separate coefficients for each level of the outcome variable, the PPO model provides a more flexible and accurate representation of the relationship between explanatory variables and crash severity. Hence, the PPO model is utilized. The PPO model distinguishes between variables whose effects can be constrained to be equal across cumulative severity thresholds and variables whose effects are allowed to vary across thresholds. The Wald chi-square test is used to assess whether the proportional-lines restriction is supported for each explanatory variable. Variables for which the restriction is not supported are specified with threshold-specific coefficients, whereas variables satisfying the restriction retain a common coefficient across thresholds. This procedure produces a restricted PPO specification that balances model flexibility with parsimony. By applying the PPO model in taxi-involved crash data, this study aims to capture the heterogeneous effects of road characteristics on different levels of crash severity and provide more meaningful insights for improving taxi-involved traffic safety.

In the study, variables can be divided into two categories. Variables satisfying the PL assumption, which is named a vector. Variables that violate the PL assumption, which is given as a vector. The Wald Chi-square tests are employed to explain whether variables satisfy the PL assumption [4,20,26]. The logit function of the PPO model is expressed as:${𝐗}_{𝐢}{𝐓}_{𝐢}$


$$\begin{array}{c} P(Y_{i}\geq j)=\frac{\text{exp}[\alpha_{j}-({\text{X}}_{\text{i}}^{\prime }\delta_{j}+{\text{T}}_{\text{i}}^{\prime }\mu_{j})]}{\text{1}+\text{exp}[\alpha_{j}-({\text{X}}_{\text{i}}^{\prime }\delta_{j}+{\text{T}}_{\text{i}}^{\prime }\mu_{j})]} \end{array}$$ (7)

where *j* means taxi-involved crash severity (i.e., slight, serious, and fatal injury). $Y_{i}$ denotes the crash injury caused by crash *i*. ${\text{X}}_{\text{i}}^{\prime }$ is the parameter with the coefficient $\delta_{j}$. ${\text{T}}_{\text{i}}^{\prime }$ denotes parameters with a coefficient $\mu_{j}$. $\alpha_{j}$ represents the threshold for the *j-*th cumulative logit.

### 4.3 Marginal effects

The marginal effects are utilized to explain the PPO model’s results since the coefficient’s sign may not always indicate explanatory variables on taxi-involved crash severity. Since all category variables in this study are dummy-coded, it is inappropriate to employ the marginal effect equation for continuous variables [45,46]. Thus, the marginal effects for taxi-involved crash *i* and severity *j* are shown as follows:


$$\begin{array}{c} E_{X_{ijk}}^{P_{ij}}=P_{ij}\left(X_{ijk}=\text{1}\right)-P_{ij}\left(X_{ijk}=\text{0}\right) \end{array}$$ (8)

where $E_{X_{ijk}}^{P_{ij}}$ denotes the marginal effects of the *k-*th dummy variable $X_{ijk}$. $P_{ij}$ is the probability of *i* with *j*. $P_{ij}\left(X_{ijk}=\text{1}\right)$ and $P_{ij}\left(X_{ijk}=\text{0}\right)$ represent the probability when $X_{ijk}$ equals 1 and 0, respectively.

## 5 Results and discussions

### 5.1 Results of the LCC

In the LCC stage, driver, environmental, and vehicle characteristics are used to identify relatively homogeneous crash contexts, whereas road characteristics are intentionally excluded from the clustering variables. This separation is adopted because the primary objective of the clustering stage is to characterize the contextual heterogeneity associated with the circumstances of taxi-involved crashes. Road characteristics are subsequently treated as the main explanatory factors of interest in the class-specific severity models. This design allows the study to examine how the same road characteristics are associated with crash severity under different crash contexts, rather than allowing road characteristics to simultaneously determine the definition of those contexts. To determine the appropriate number of classes, the LCC involves three uncontrolled categories through Mplus 8.0 software (i.e., driver, environment, and crash characteristics), as exhibited in Table 2. The number of classes in this study is set to range from 1 to 10, considering pertinent studies [20,42,43]. Fig 1 and Table 3 illustrate how the Entropy value and the three criteria (i.e., AIC, BIC, and ABIC) vary.

**Table 3:** The estimated results of four indices.

Class	Value			Reduction rate/%			Entropy
	AIC	BIC	ABIC	AIC	BIC	ABIC	
1	113734	113734	113734	–	–	–	1.000
2	108267	109361	108933	4.81	3.85	4.22	0.886
3	106210	107501	107071	1.90	1.70	1.71	0.834
4	104879	106211	105775	1.25	1.20	1.21	0.853
5	103871	105256	104729	0.96	0.90	0.99	0.914
6	102847	104549	103891	0.99	0.67	0.80	0.882
7	102127	103996	103268	0.70	0.53	0.60	0.847
8	101616	103684	102854	0.50	0.30	0.40	0.853
9	101213	103580	102649	0.40	0.10	0.20	0.836
10	101109	103580	102546	0.10	0.00	0.10	0.871

**Fig 1:**
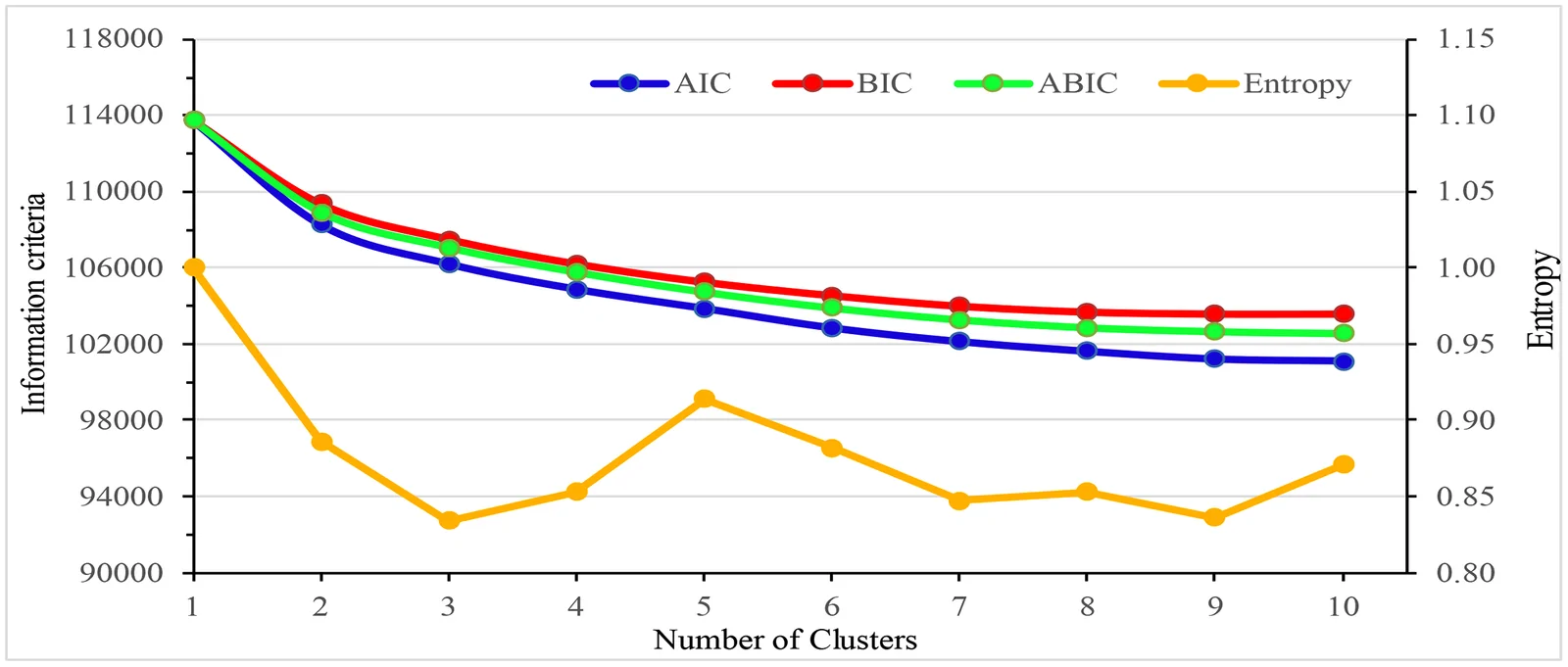
The evolution of the Entropy value and three criteria for the number of classes.

As shown in Fig 1 and Table 3, the Entropy value at the five classes exceeds 0.9. Three criteria (i.e., AIC, BIC, and ABIC) decrease accordingly as the number of classes increases. However, the minimum values are not reached in all tested models. Therefore, the number of classes is identified using the reduction percentage of the three criteria [43]. The reduction percentage of the three criteria from five classes afterward falls below 1%. These results indicate that the five classes are an appropriate segment for taxi-involved crash data.

Because of the maximum homogeneity within each class and heterogeneity between the five classes, the five classes can be characterized according to the skewed feature of special variables [20,42]. For example, 90% of the crashes in one class result from adverse weather (e.g., rain and snow), while more balanced distributions exist in the feature of “weather” that falls into other classes. The class can be referred to as “adverse weather traffic crashes”. Notably, the analysis is aimed at identifying the types of crash data, which might overlap on several variables.

The five classes are identified through the LCC analysis, allowing for the characterization of distinct patterns within the taxi-involved crash data [20,42,43]. Table 4 details the skewed distribution of special variables among the five classes. Class 1 is characterized by a high occurrence of crashes in females (99.0%) and a significant proportion caused by elderly drivers (65+) (62.9%). Therefore, it is classified as “Crashes involving females caused by elderly drivers (65+)”. Characterized by a high proportion of crashes caused by drivers aged 35–64 (84.5%), class 2 involves non-commuting trips only (100.0%), for which it is designated as “Crashes involving non-commuting trips caused by drivers aged 35 to 64”. Class 3 is characterized by crashes exclusively caused by males (100.0%), and it occurs significantly in fine weather conditions (86.3%). Therefore, it is classified as “Crashes involving males in fine weather”. Class 4 is characterized by a high occurrence of crashes on weekends (96.6%), with a significant proportion occurring at night (88.1%). For this reason, it is designated as “Crashes occurring on weekends at night”. Class 5 is characterized by a high proportion of crashes caused by females (94.4%), with a significant occurrence among young drivers (24-) (57.3%), for which it is classified as “Crashes involving females among young drivers (24-)”.

**Table 4:** The five crash classes identified by the special variables.

Variable		Percentage/%				
		Class 1	Class 2	Class 3	Class 4	Class 5
Taxi-involved crashes		16.34	15.88	40.40	11.28	16.10
Gender	Male	1.0	81.1	**100.0**	76.3	5.6
	Female	**99.0**	20.9	0.0	23.7	**94.4**
Age	24-	1.5	1.6	2.0	0.6	**57.3**
	35-64	28.1	**84.5**	74.4	76.1	35.7
	65+	**62.9**	7.6	9.0	6.3	1.1
Travel purpose	Non commuting	63.0	**100.0**	23.5	27.7	30.1
Day	Weekend	28.5	28.7	22.4	**96.6**	24.0
Time	Night	37.8	44.1	37.2	**88.1**	36.9
Weather	Fine	79.5	73.8	**86.3**	76.5	73.3

It should be noted that these class labels are descriptive representations of the dominant characteristics observed within each latent class rather than causal classifications. The classes are identified statistically according to the joint distribution of the selected driver, environmental, and vehicle characteristics. Therefore, a class should be interpreted as a relatively homogeneous crash context rather than as a single underlying cause of crashes. In addition, because several characteristics may be shared across classes, the five crash profiles should not be interpreted as completely mutually exclusive behavioral or environmental mechanisms.

### 5.2 Results of the PPO model

A restricted PPO model is first constructed to compare the base model with the five sub-classes presented through Stata 15 software, involving 9788 taxi-involved crash records. The log-likelihood value at convergence is −3558.797. Then, PPO models are presented for the five classes. The sum of the log-likelihood values for the five classes is −3478.022. The value of test statistic is 213.17 with 26 degrees of freedom, indicating the appropriateness of the five classes. The road characteristics conforming to the PL assumption exert consistent and constant effects, while those violating the PL assumption have diverse effects on different severities of taxi-involved crashes. Additionally, the multi-collinearity is checked through the variance inflation factor (VIF). All VIF values were lower than 5.0, demonstrating that the multi-collinearity satisfies the requirements [42,47]. Tables 5–9 present the results of the five PPO models accordingly.$\chi^{\text{2}}$

**Table 5:** PPO model for Class 1 in taxi-involved crashes.

Variable	All Level ^k^		Slight-Serious		Serious-Fatal	
	Coef.	z-value	Coef.	z-value	Coef.	z-value
Intercept			−1.715	−2.10**	−4.347	−7.12***
*Road class (vs. arterial road and motorway)*						
Sub-arterial road			−0.263	−1.77*	−0.253	−1.83*
*Road type (vs. roundabout)*						
Single carriageway			0.569	2.16**	0.440	2.05**
Dual carriageway	−0.372	−1.75*				
*Speed limit (vs. ≤ 20 mph)*						
31-40 mph	0.839	1.94*				
≥40 mph			1.341	2.09**	1.451	2.69***
*Junction detail (vs. no junction in 20m)*						
Crossroads			−0.262	−1.83*	−0.181	−1.72*
Junction with more than 4 arms			−0.254	−1.94*	−0.284	−1.98**
*Junction Control (vs. not applicable)*						
Traffic signal			−0.582	−2.17**	−0.651	−2.33**
Stop sign/give way			−0.351	−1.67*	−0.265	−1.77*
*Pedestrian crossing (vs. no in 50 meters)*						
Pedestrian phase at traffic signal junction			0.495	2.19**	0.182	1.89*
*Light condition (vs. daylight)*						
Dark			−0.103	−1.83*	−0.121	−1.86*
*Road surface (vs. dry)*						
Ice/snow			−1.263	−1.71*	−1.614	−1.97**

^k^Variables obey the PL assumption. No. of observations = 1596. Log-likelihood (null)=−521.056. Log-likelihood (model)=−472.929. AIC = 993.859, BIC = 1122.866. * Level of significance>90%, ** Level of significance>95%, *** Level of significance>99%.

**Table 6:** PPO model for Class 2 in taxi-involved crashes.

Variable	All Level ^k^		Slight-Serious		Serious-Fatal	
	Coef.	z-value	Coef.	z-value	Coef.	z-value
Intercept	−6.646	−7.03***				
*Road class (vs. arterial road and motorway)*						
Sub-arterial road	−0.092	−1.68*				
*Speed limit (vs.* ≤*20 mph)*						
21-30 mph	−0.137	−1.79*				
31-40 mph	1.169	1.85*				
≥40 mph			1.373	1.67*	2.843	2.38**
*Junction detail (vs. no junction in 20m)*						
Junction with more than 4 arms			0.278	1.98**	0.202	1.90*
*Junction Control (vs. not applicable)*						
Traffic signal			−0.734	−2.08**	−0.238	−1.81*
*Pedestrian crossing (vs. no in 50 meters)*						
Pedestrian phase at traffic signal junction	0.483	2.04**				
*Light Condition (vs. daylight)*						
Dark	0.192	1.78*				
*Road surface (vs. dry)*						
Wet	−0.109	−1.76*				
*Restricted lane (vs. unrestricted lane)*						
Motorized vehicle lanes	−2.092	−4.51***				

^k^Variables obey the PL assumption. No. of observations = 1554. Log-likelihood (null)=−588.558. Log-likelihood (model)=−550.410. AIC = 1148.821, BIC = 1277.188. * Level of significance>90%, ** Level of significance>95%, *** Level of significance>99%.

**Table 7:** PPO model for Class 3 in taxi-involved crashes.

Variable	All Level ^k^		Slight-Serious		Serious-Fatal	
	Coef.	z-value	Coef.	z-value	Coef.	z-value
Intercept	−5.794	−7.41***				
*Road class (vs. arterial road and motorway)*						
Sub-arterial road	−0.277	−1.66*				
Side way	−0.215	−1.87*				
*Road type (vs. roundabout)*						
One-way street	−0.231	−1.66*				
Dual carriageway	−0.297	−1.72*				
*Speed limit (vs. ≤ 20 mph)*						
31-40 mph			−0.029	−1.70*	−0.036	−1.88*
≥40 mph			0.142	2.37**	0.226	2.54**
*Junction Control (vs. not applicable)*						
Traffic signal	−3.168	−2.74***				
Stop sign/give way			0.433	1.94*	−2.897	−2.34**
*Pedestrian crossing (vs. no in 50 meters)*						
Pedestrian phase at traffic signal junction	0.187	1.68*				
*Light Condition (vs. daylight)*						
Dark	0.107	1.98**				
*Road surface (vs. dry)*						
Wet	−0.222	−1.77*				
Ice/snow	−1.391	−2.35**				
*Restricted lane (vs. unrestricted lane)*						
Motorized vehicle lanes			−0.546	−2.14**	−0.426	−1.99**
Non-motorized vehicle lanes	0.475	1.76*				

^k^Variables obey the PL assumption. No. of observations = 3954. Log-likelihood (null)= −1559.655. Log-likelihood (model)=−1523.481. AIC = 3104.97, BIC = 3287.164. * Level of significance>90%, ** Level of significance>95%, *** Level of significance>99%.

**Table 8:** PPO model for Class 4 in taxi-involved crashes.

Variable	All Level ^k^		Slight-Serious		Serious-Fatal	
	Coef.	z-value	Coef.	z-value	Coef.	z-value
Intercept	−6.818	−6.97***				
*Road class (vs. arterial road and motorway)*						
Sub-arterial road	0.398	1.84*				
Side way	−0.146	−1.66*				
*Road type (vs. roundabout)*						
Single carriageway	0.871	2.15**				
Dual carriageway	0.962	2.23**				
*Speed limit (vs. ≤ 20 mph)*						
31-40 mph	0.027	1.75*				
≥40 mph	−0.044	−1.92*				
*Junction detail (vs. no junction in 20m)*						
Roundabout	−0.056	−1.79*				
Crossroads	−0.638	−2.28**				
Junction with more than 4 arms			−1.07	−2.52**	−0.741	−2.02**
*Junction Control (vs. not applicable)*						
Traffic signal	−0.160	−1.93*				
*Pedestrian crossing (vs. no in 50 meters)*						
Zebra/pelican/central refuge	0.498	2.08**				
Pedestrian phase at traffic signal junction			0.690	2.41**	0.310	2.01**
*Light condition (vs. daylight)*						
Dark	0.572	2.74***				
*Road surface (vs. dry)*						
Wet	−0.204	−1.91*				
Ice/snow	−0.616	−2.81***				

^k^Variables obey the PL assumption. No. of observations = 1104. Log-likelihood (null)= −424.075. Log-likelihood (model)= −380.345. AIC = 806.691, BIC = 921.845. * Level of significance>90%, ** Level of significance>95%, *** Level of significance>99%.

**Table 9:** PPO model for Class 5 in taxi-involved crashes.

Variable	All Level ^k^		Slight-Serious		Serious-Fatal	
	Coef.	z-value	Coef.	z-value	Coef.	z-value
Intercept	−8.087	−6.38***				
*Road class (vs. arterial road and motorway)*						
Sub-arterial road	−0.386	−2.14**				
Side way			−0.304	−1.97**	−0.114	−1.66*
*Road type (vs. roundabout)*						
One-way street	0.176	1.68*				
Single carriageway	0.777	2.84***				
Dual carriageway	0.272	1.93*				
*Speed limit (vs. ≤ 20 mph)*						
≥40 mph	0.339	2.51**				
*Junction detail (vs. no junction in 20m)*						
Roundabout	−0.172	−1.77*				
T/staggered junction	−0.578	−2.16**				
Crossroads	−1.076	−2.13**				
Junction with more than 4 arms			−0.201	−1.84*	−0.362	−1.97**
*Junction Control (vs. not applicable)*						
Traffic signal	0.240	1.76*				
*Pedestrian crossing (vs. no in 50 meters)*						
Pedestrian phase at traffic signal junction	0.028	2.38**				
*Light condition (vs. daylight)*						
Dark	0.148	1.92*				
*Road surface (vs. dry)*						
Wet	−0.428	−1.88*				

^k^Variables obey the PL assumption. No. of observations = 1580. Log-likelihood (null)=−602.554. Log-likelihood (model)=−582.315. AIC = 1210.632, BIC = 1334.031. * Level of significance>90%, ** Level of significance>95%, *** Level of significance>99%.

There are significant variations in road characteristics across the five classes, as observed in different severities of taxi-involved crashes, which underscores the importance attached to revealing the heterogeneity in crash data through the LCC. Despite being in the same class, the parameters of the same variables differ between different crash severities. These results show the presence of significant heterogeneity within and across the five classes. Notably, what indicates the effects of the intermediate results is not limited to the sign(s) of the coefficient(s) [45]. Because the explanatory variables are categorical, the reported marginal effects represent the change in the predicted probability of each crash severity category associated with changing a categorical variable from its reference category to the specified category, while accounting for the other variables included in the model. Therefore, a positive marginal effect indicates an increase in the predicted probability of the corresponding severity category relative to the reference condition, whereas a negative value indicates a decrease. Thus, it is necessary to calculate the marginal effects of each significant variable to understand how road characteristics affect the severity of taxi-involved crashes and to identify the differences among the five classes. Tables 10–14 show the variations in the effects of significant road characteristics among the five classes.

**Table 10:** The marginal effects of road characteristics for Class 1.

Variable	Slight		Serious		Fatal	
	%	95% c.i.	%	95% c.i.	%	95% c.i.
*Road class (vs. arterial road and motorway)*						
Sub-arterial road	1.20	(−2.14,4.55)	−1.13	(−4.31,2.03)	−0.07	(−0.24,0.11)
*Road type (vs. roundabout)*						
Single carriageway	−4.20	(−13.33,4.90)	3.58	(−4.28,11.44)	0.82	(−0.74,1.98)
Dual carriageway	−3.92	(−11.03,3.15)	3.18	(−2.30,8.61)	0.74	(−1.07,2.58)
*Speed limit (vs. ≤ 20 mph)*						
31-40 mph	−8.73	(−19.61,2.15)	8.27	(−2.02,18.56)	0.46	(−0.22,1.15)
≥40 mph	−15.69	(−35.57,4.11)	14.76	(−3.73,33.26)	0.93	(−0.61,2.46)
*Junction detail (vs. no junction in 20m)*						
Crossroads	7.44	(−1.21,14.38)	−6.99	(−14.07,1.06)	−0.45	(−1.37,0.28)
Junction with more than 4 arms	1.66	(−6.01,9.33)	−1.57	(−8.83,5.69)	−0.09	(−0.50,0.32)
*Junction Control (vs. not applicable)*						
Traffic signal	9.06	(−8.58, 28.41)	−8.56	(−31.79,15.91)	−0.50	(−2.69,1.69)
Stop sign/give way	8.41	(−5.01,21.31)	−7.93	(−20.97,5.11)	−0.48	(−1.43,0.45)
*Pedestrian crossing (vs. no in 50 meters)*						
Pedestrian phase at traffic signal junction	1.69	(−2.57,5.19)	−1.60	(−4.07,1.28)	−0.09	(−0.52,0.30)
*Light condition (vs. daylight)*						
Dark	0.79	(−2.11,3.60)	−0.74	(−3.01,1.98)	−0.05	(−0.20,0.01)
*Road surface (vs. dry)*						
Wet	6.73	(2.13,11.32)	−6.39	(−10.71,-2.11)	−0.34	(−0.64,-0.01)

Note: The first category for each variable, seen in the brackets, is regarded as the base.

**Table 11:** The marginal effects of road characteristics for Class 2.

Variable	Slight		Serious		Fatal	
	%	95% c.i.	%	95% c.i.	%	95% c.i.
*Road class (vs. arterial road and motorway)*						
Sub-arterial road	0.91	(−4.89,6.71)	−0.89	(−6.55,4.77)	−0.02	(−0.05, 0.01)
Speed limit (vs. ≤ 20 mph)						
21-30 mph	−1.38	(−4.93,2.12)	−0.02	(−0.10,0.04)	1.40	(−2.10,5.00)
31-40 mph	−1.37	(−2.33,-0.41)	−0.15	(−0.30,0.05)	1.52	(0.45,2.59)
≥40 mph	−2.82	(−15.85,10.21)	1.21	(−12.11,14.63)	1.61	(0.62,2.57)
*Junction detail (vs. no junction in 20m)*						
Junction with more than 4 arms	2.32	(−3.78,7.61)	−2.01	(−4.52,2.53)	−0.31	(−0.61,0.26)
*Junction Control (vs. not applicable)*						
Traffic signal	9.41	(3.71,15.11)	−9.21	(−14.79,-3.64)	−0.20	(−0.42,0.03)
*Pedestrian crossing (vs. no in 50 meters)*						
Pedestrian phase at traffic signal junction	5.03	(−0.23,10.15)	−4.90	(−9.14,0.95)	−0.13	(−0.66,0.32)
*Light condition (vs. daylight)*						
Dark	−1.95	(−5.23,1.31)	1.90	(−1.28,5.10)	0.05	(−0.04,0.14)
*Road surface (vs. dry)*						
Ice/snow	1.09	(−2.66,4.85)	−1.06	(−4.73,2.60)	−0.03	(−0.12,0.06)
*Restricted lane (vs. unrestricted lane)*						
Motorized vehicle lanes	11.80	(9.22,14.41)	−11.55	(−14.11,-9.02)	−0.25	(−0.52,0.01)

Note: The first category for each variable, seen in the brackets, is regarded as the base.

**Table 12:** The marginal effects of road characteristics for Class 3.

Variable	Slight		Serious		Fatal	
	%	95% c.i.	%	95% c.i.	%	95% c.i.
*Road class (vs. arterial road and motorway)*						
Sub-arterial road	2.93	(−0.71,6.58)	−2.86	(−6.43,0.70)	−0.07	(−0.09, 0.21)
Side way	2.33	(−0.04,4.71)	−2.20	(−4.61,0.04)	−0.13	(−0.11,0.01)
*Road type (vs. roundabout)*						
One-way street	−2.68	(−6.78,1.93)	2.36	(−1.35,6.98)	0.32	(−1.18,2.20)
Dual carriageway	−3.08	(−9.10,3.28)	3.01	(−2.95,8.99)	0.07	(−0.15,0.29)
*Speed limit (vs. ≤ 20 mph)*						
31-40 mph	−0.31	(−5.71,4.34)	0.30	(−6.21,6.80)	0.01	(−0.14,0.17)
≥40 mph	−1.61	(−10.53,7.29)	1.58	(−7.12,10.28)	0.03	(−0.17,0.25)
*Junction Control (vs. not applicable)*						
Traffic signal	9.03	(−14.19,12.13)	−6.57	(−6.78,7.93)	−2.46	(−5.41,6.33)
Stop sign/give way	5.19	(−4.16,5.38)	−3.79	(−4.33,11.93)	−1.40	(−12.19,3.37)
*Pedestrian crossing (vs. no in 50 meters)*						
Pedestrian phase at traffic signal junction	2.29	(−0.58,5.91)	−2.24	(−5.97,0.46)	−0.05	(−0.13,0.02)
*Light condition (vs. daylight)*						
Dark	−1.18	(−3.35,0.98)	1.16	(−0.95,3.28)	0.02	(−0.02,0.07)
*Road surface (vs. dry)*						
Wet	9.70	(5.35,14.05)	−9.56	(−13.85,-5.27)	−0.14	(−0.26,-0.01)
Ice/snow	2.72	(−0.18,5.20)	−2.36	(−4.12,0.85)	−0.36	(−0.89,0.12)
*Restricted lane (vs. unrestricted lane)*						
Motorized vehicle lanes	4.96	(1.24,8.67)	−4.85	(−8.49,-1.21)	−0.11	(−0.21,0.01)
Non-motorized vehicle lanes	6.24	(−4.23,16.74)	−6.08	(−14.69,1.86)	−0.16	(−0.67,0.38)

Note: The first category for each variable, seen in the brackets, is regarded as the base.

**Table 13:** The marginal effects of road characteristics for Class 4.

Variable	Slight		Serious		Fatal	
	%	95% c.i.	%	95% c.i.	%	95% c.i.
*Road class (vs. arterial road and motorway)*						
Sub-arterial road	4.49	(3.96, 4.94)	−4.31	(−9.78,-1.41)	−0.18	(−0.45,0.06)
Side way	1.30	(−3.07,5.83)	0.03	(−0.21,0.11)	−1.33	(−5.63,2.96)
*Road type (vs. roundabout)*						
Single carriageway	−7.42	(−16.45,1.60)	7.10	(−1.56,15.92)	0.32	(−0.12,0.62)
Dual carriageway	−6.48	(−14.71,1.73)	6.27	(−1.71,14.24)	0.21	(−0.10,0.53)
*Speed limit (vs. ≤ 20 mph)*						
31-40 mph	−0.28	(−10.87,10.31)	0.27	(−9.92,10.40)	0.01	(−0.31,0.41)
≥40 mph	−0.14	(−15.91,15.62)	0.10	(−6.22,6.51)	0.04	(−0.61,0.68)
*Junction detail (vs. no junction in 20m)*						
Roundabout	0.67	(−14.61,15.95)	−0.65	(−15.33,14.02)	−0.02	(−0.62,0.57)
Crossroads	6.47	(−1.21,14.16)	−6.24	(−13.65,1.17)	−0.23	(−0.59,0.12)
Junction with more than 4 arms	8.46	(2.71,14.23)	−8.14	(−13.66,-2.61)	−0.32	(−0.72,0.07)
*Junction Control (vs. not applicable)*						
Traffic signal	12.18	(6.57,18.20)	−11.68	(−27.01,4.38)	−0.50	(−3.26,2.25)
*Pedestrian crossing (vs. no in 50 meters)*						
Zebra/pelican/central refuge	4.83	(0.63,9.01)	−4.66	(−9.03,0.28)	−0.17	(−0.46,0.01)
Pedestrian phase at traffic signal junction	7.14	(1.47,14.81)	−6.87	(−12.79,1.95)	−0.27	(−0.69,0.13)
*Light condition (vs. daylight)*						
Dark	−5.44	(−9.18,-1.70)	5.25	(1.63,8.87)	0.19	(−0.03,0.42)
*Road surface (vs. dry)*						
Wet	5.19	(−4.94,15.33)	−5.01	(−14.83,4.81)	−0.18	(−0.53,0.18)
Ice/snow	1.90	(−2.90,5.09)	−1.00	(−2.16,0.12)	−0.90	(−0.33,0.11)
*Restricted lane (vs. unrestricted lane)*						

Note: The first category for each variable, seen in the brackets, is regarded as the base.

**Table 14:** The marginal effects of road characteristics for Class 5.

Variable	Slight		Serious		Fatal	
	%	95% c.i.	%	95% c.i.	%	95% c.i.
*Road class (vs. arterial road and motorway)*						
Sub-arterial road	0.41	(−5.21,6.02)	−0.40	(−5.99,5.17)	−0.01	(−0.05, 0.06)
Side way	0.32	(−3.53,4.10)	−0.30	(−4.16,3.50)	−0.02	(−0.02,0.03)
*Road type (vs. roundabout)*						
One-way street	−5.42	(−13.34,2.49)	5.38	(−2.48,12.26)	0.04	(−0.04,0.11)
Single carriageway						
Dual carriageway	−5.82	(−15.34,3.69)	5.79	(−3.60,15.26)	0.03	(−0.04,0.11)
*Speed limit (vs. ≤ 20 mph)*						
≥40 mph	−4.12	(−21.53,13.27)	4.10	(−13.19,21.39)	0.02	(−0.09,0.15)
*Junction detail (vs. no junction in 20m)*						
Roundabout	6.57	(−4.17,17.32)	−6.53	(−17.21,4.15)	−0.04	(−0.12,0.02)
T/staggered junction	9.69	(−1.02,19.61)	−9.69	(−25.56,7.95)	0.00	(−0.04,0.05)
Crossroads	8.64	(0.61,16.69)	−8.60	(−16.61,-0.59)	−0.04	(−0.14,0.05)
Junction with more than 4 arms	2.20	(−6.05,10.48)	−2.19	(−10.41,6.01)	−0.01	(−0.06,0.04)
*Junction Control (vs. not applicable)*						
Traffic signal	11.21	(−8.31,35.86)	−11.14	(−25.71,8.03)	−0.07	(−0.37,0.11)
*Pedestrian crossing (vs. no in 50 meters)*						
Pedestrian phase at traffic signal junction	3.18	(1.91,5.54)	−3.16	(−7.53,1.86)	−0.02	(−0.15,0.08)
*Light condition (vs. daylight)*						
Dark	−1.61	(−5.09,1.87)	1.60	(−1.86,5.06)	0.01	(−0.01,0.03)
*Road surface (vs. dry)*						
Wet	4.23	(0.26,8.19)	−4.20	(−8.14,-0.20)	−0.02	(0.01,0.02)

Note: The first category for each variable, seen in the brackets, is regarded as the base.

#### 5.2.1 *Basic attributes.*

The basic attributes include road class, road type, speed limit, and restricted lane. In terms of road class, sub-arterial roads are less likely to cause severe injuries (i.e., serious and fatal injuries) in most classes, with the highest possibility observed in Class 4 (crashes occurring on weekends at night) (marginal effects: −4.31% for serious injury and −0.18% for fatal injury). Side-ways show a lower probability of causing severe injuries in Class 3, Class 4, and Class 5, with the highest probability observed in Class 3 (marginal effects: −2.20% for serious injury and −0.13% for fatal injury). Importantly, side-ways increase the risks of serious injury in Class 4 (marginal effects: + 0.03%), which is potentially attributable to the occurrence of nighttime crashes in Class 4. This could result from the lower travel speeds of vehicles on low-grade roads, which is conducive to reducing crash severity when an accident occurs. To enhance safety in taxi-involved environments on high-grade roads, it is advisable to impose speed limits as appropriate, which depends on the exact road function and grade.

Regarding road type, one-way streets are more likely to cause serious injury in Class 3 (marginal effects: + 2.36%) and Class 5 (marginal effects: + 5.38%), compared to roundabouts. Single carriageways tend to heighten the risk of serious injury in Class 1 (crashes involving females caused by elderly drivers (65+)) (marginal effects: + 3.58%) and Class 4 (marginal effects: + 7.10%). Dual carriageways show a greater possibility of causing serious injury in Class 1 (marginal effects: + 3.18%), Class 3 (marginal effects: + 3.01%), Class 4 (marginal effects: + 6.27%), and Class 5 (marginal effects: + 5.79%). This results from the higher vehicle speeds on one-way streets, single carriageways, and dual carriageways due to their simpler traffic environments, compared to the roundabouts where vehicles travel at lower speeds. Importantly, in Class 3, dual carriageways (marginal effects: + 3.01%) are more likely to cause serious injury than one-way streets (marginal effects: + 2.36%). According to these estimation results, it is crucial to separate two-way traffic and mitigate the severity of taxi-involved crashes by installing preventative facilities (e.g., green belts).

With respect to the speed limit, the speed limits between 21–30 mph lead to the heightened risk of fatal injury in Class 2 (crashes with non-commuting trips caused by drivers from 35 to 64 years old) (marginal effects: + 1.40%), compared to speed limits within 20 mph. Notably, the speed limits between 21–30 mph have a lower probability of causing serious injury (marginal effects: −0.02%). This is possibly attributed to the fact that the crashes in Class 2 involve middle-aged drivers (35–64) who are typically more experienced in driving. The speed limits between 31–40 mph increase the likelihood of occurrence for severe injuries in Class 1 (marginal effects: + 8.27% for serious injury), Class 2 (marginal effects: + 1.52% for fatal injury), Class 3 (marginal effects: + 0.30% for serious injury), and Class 4 (marginal effects: + 0.27% for serious injury). Notably, speed limits over 40 mph are more likely to cause severe injuries in all five classes, with the highest likelihood observed in Class 1 (marginal effects: + 14.76% for serious injury). Therefore, it is necessary to carefully consider avoiding speed limits of over 30 mph for taxis.

With regard to restricted lanes, in Class 2, motorized vehicle lanes are associated with a lower likelihood of severe injuries, with the marginal effects reaching −11.55% for serious injury and −0.25% for fatal injury, respectively. Similar trends are observable in Class 3, with the marginal effects reaching −4.85% for serious injury and −0.11% for fatal injury, respectively. Similarly, non-motorized vehicle lanes are linked to a reduced likelihood of severe injuries in Class 3, with the marginal effects reaching −6.08% for serious injury and −0.16% for fatal injury, respectively. Overall, these findings indicate that restricted lanes may contribute to lowering the probability of severe injuries in taxi-involved crashes. This is potentially attributable to the separation of different types of traffic in restricted lanes. Since prior studies have led to similar results [48], it is crucial to avoid mixed traffic and enhance road safety by establishing clear right-of-way rules for different traffic modes.

#### 5.2.2 *Junction attributes.*

Junction attributes include junction details, junction control, and pedestrian crossing facilities. Junction details tend to have a significant impact on taxi-involved crash severity. Compared to the absence of junctions within a 20m distance, roundabouts are less likely to cause severe injuries in Class 4 and Class 5, particularly in Class 5 (marginal effects: −6.53% for serious injury). T/staggered junctions lead to a significant reduction in the probability of serious injury in Class 5 (marginal effects: −9.69%). Crossroads mitigate the risks of serious injury in Class 1 (marginal effects: −6.99%), Class 4 (marginal effects: −6.24%), and Class 5 (marginal effects: −8.60%). The junctions with more than four arms are less likely to cause serious injury in all five classes, with a significant effect observed in Class 4 (marginal effects: −8.14%). According to these estimations, the likelihood of severe injuries is significantly reduced by junctions. Notably, T/staggered junctions exhibit the most significant marginal effects compared to roundabouts, crossroads, and junctions with more than four arms. In general, the severity of taxi-involved crashes declines with a reduction in the number of arms at junctions (except for roundabouts). It is essential to enhance road safety by reducing the number of arms at junctions for the design of road layouts.

Junction control exerts a positive effect on the mitigation of taxi-involved crash severity compared to junctions without control. Traffic signals mitigate the risks of serious injury in all five classes. The marginal effects for serious injury are −8.56%, −9.21%, −6.57%, −11.68%, and −11.14% in Class 1, Class 2, Class 3, Class 4, and Class 5, respectively. Additionally, these controls (stop signs and give ways) reduce the risk of serious injury in Class 1 (marginal effects: −7.93%). A potential reason for this is that the presence of proper controls (e.g., traffic signals, stop signs, and give ways) at intersections reduces the conflicts between taxis and other road users, such as pedestrians and cyclists. To reduce the likelihood of severe injuries in taxi-involved crashes, it is necessary to take into account traffic volume and safety impacts when the need for junction controls is determined, particularly traffic signals.

Pedestrian crossing facilities are another significant influencing factor for the severity of taxi-involved crashes. In Class 4, the likelihood of severe injuries is reduced by the presence of flexible pedestrian crossing facilities (e.g., zebra crossings, pelican crossings, and central refuges) within a 50m range, with the marginal effects reaching −4.66% for serious injury and −0.17% for fatal injury, respectively. Traffic signals are less likely to cause serious injury in all five classes, with the marginal effects reaching −1.60%, −4.90%, −2.24%, −6.87%, and −3.16% for serious injury in Class 1, Class 2, Class 3, Class 4, and Class 5, respectively. These findings suggest that pedestrian crossing facilities contribute to mitigating the severity of taxi-involved crashes. Notably, traffic signals exert a more significant positive impact than flexible facilities on reducing severe injuries. This is possible because the conflicts between motorized and non-motorized vehicles are mitigated by requiring drivers to stop at the junctions with traffic signals when the lights are red. The proper installation of traffic signals at intersections plays a crucial role in determining the right-of-way during conflicts between motor vehicles and other modes, which enhances taxi-involved travel safety. There is a possibility that the likelihood of taxi-involved crashes is mitigated by the installation of automated traffic signals or the presence of authorized personnel.

#### 5.2.3 *Road conditions.*

Road conditions mainly include road surface and light conditions. Regarding light conditions, darkness is associated with a higher risk of severe injuries compared to daylight in Class 2, with the marginal effects reaching +1.90% for serious injury and +0.05% for fatal injury, respectively. Similar patterns are observable in Class 3, Class 4, and Class 5, with the most pronounced effects observed in Class 4 (+5.25% for serious injury and +0.19% for fatal injury). Notably, darkness contributes to a slight reduction in severe injuries in Class 1, with the marginal effects reaching −0.74% for serious injury and −0.05% for fatal injury, respectively. One possible explanation is that the characteristics of Class 1 differ substantially from those of the other classes, particularly in terms of driver composition. However, the present dataset does not directly measure driving behavior or risk perception, and therefore this interpretation should be regarded as a possible explanation rather than a causal mechanism. Therefore, it is significant to reduce the likelihood of severe injuries in taxi-involved crashes by improving lighting conditions. It is thus recommended to install street lights for better lighting conditions and greater road safety.

Regarding road surface, in Class 1, wet road surfaces contribute to a lower likelihood of severe injuries, with the marginal effects reaching −6.39% for serious injury and −0.34% for fatal injury, respectively. Similar trends are observable in Class 3, Class 4, and Class 5, with the highest marginal effects observed in Class 3 (−9.56% for serious injury). Similarly, other adverse road surfaces, such as ice and snow, are linked to a reduced likelihood of severe injuries in Class 2, with the marginal effects reaching −1.06% for serious injury and −0.03% for fatal injury, respectively. Similar patterns can be found in Class 3 and Class 4, with the highest impacts observed in Class 3 (−2.36% for serious injury). Overall, it is suggested by these findings that adverse road surfaces can lower the likelihood of severe injuries in taxi-involved crashes. One possible explanation is that adverse weather or road-surface conditions may be associated with lower travel speeds or different driving patterns, which could reduce injury severity conditional on a crash. Additionally, wet road surfaces may be more likely to mitigate severe injuries than ice and snow road surfaces, which is potentially due to improved traction. Similar results were observed in Wang et al. [42], which emphasized that poor surface conditions resulted in ice and snow, leading to more severe injuries than rain for taxi severity.

## 6 Conclusion

This study aims to demonstrate the heterogeneous effects of road characteristics on different levels of taxi-involved crash severity. Considering uncontrolled factors (i.e., driver, weather, and vehicle factors), taxi-involved crash data were divided into five distinct classes through the LCC. These five distinct classes are characterized and represented by different skewed features. Allowing for the hierarchical nature of crash severities, the PPO model was used to determine the heterogeneous effects of road characteristics on different crash scenarios within each class. Besides, marginal effects were measured to further reveal the significant effects of road characteristics.

The results unravel the varying roles of road characteristics across different classes, with certain road characteristics of the same class exhibiting diverse coefficients in relation to different crash severities. Regarding different classes, such as Class 4, junction details are less likely to cause severe injuries, whereas no significant effect is observable in Class 1. Within the same class, such as Class 2, motorized vehicle lanes are associated with a lower risk of severe injuries, whereas no significant correlations are observed for non-motorized vehicle lanes. These findings demonstrate the diverse impacts of road characteristics on crash severities, both within and across the five classes, providing valuable insights into mitigating the severity of taxi-involved crashes.

A range of road characteristics contribute to the low risk of severe injuries in taxi-involved crashes, such as low-grade roads, roads with isolation facilities, lower speed limits, junctions with fewer arms, intersections with proper controls, pedestrian crossing facilities within 50m, daylight, adverse road surfaces, and restricted lanes. There are three most significant road characteristics that affect crash severities: speed limits, junction controls, and restricted lanes. In terms of speed limits, the level of crash severity rises when speed limits are higher. Particularly, the speed limits exceeding 40 mph have the most significant impact on crash severity. Therefore, it is crucial to mitigate taxi-involved crash severity by thoroughly evaluating the speed limits of over 40 mph for taxis. Furthermore, junction control is a significant factor that affects the level of crash severity. The presence of controls, such as traffic signals, stop signs, and give ways, is conducive to reducing crash severity. Among them, traffic signals have the most pronounced impact, with serious injuries reduced by −11.68%. Therefore, it is essential to enforce appropriate controls at junctions for the enhanced safety of taxi-involved travel. Unrestricted lanes play a crucial role as well, and motorized vehicle lanes mitigate the risk of severe injuries significantly (−11.55%). These findings provide valuable and meaningful insights into the diverse impacts of road characteristics, which contribute to improving taxi-involved travel safety. Importantly, the heterogeneous effects identified in this study suggest that road safety interventions should not necessarily be implemented uniformly across all taxi-involved crashes. For example, speed management may be particularly relevant to crash contexts characterized by higher-speed road environments, whereas junction control and pedestrian facilities may deserve greater attention in contexts involving substantial interaction between taxis and other road users. Similarly, lighting and road-surface interventions may be prioritized according to the crash contexts in which these factors exhibit stronger associations with injury severity. Thus, the latent classes provide a useful basis for developing more context-specific safety strategies for taxi operations.

In summary, this study highlights the importance of exploring the heterogeneous effects of road characteristics on taxi-involved crash severity through the LCC and PPO models. It can be improved by accomplishing the following research tasks. Firstly, there might be variations in the heterogeneous effects of road characteristics over time, indicating the temporal instability in taxi-involved crash data [49, 50]. Therefore, future research can pay attention to examining the temporal stability and generalizability of taxi-involved crash data. Secondly, crashes are often rare events, which leads to an imbalance in the data between fatal injury, serious injury, and slight injury. Such data imbalance has been widely reported in numerous previous studies [42]. It can be taken into consideration in future research. Thirdly, the empirical analysis is based on taxi-involved crash data from London, and the identified crash profiles and road-characteristic associations may depend on the local road network, traffic regulations, taxi operating environment, and travel characteristics. Therefore, caution should be exercised when generalizing the findings to other cities or countries. Future studies could validate the proposed framework using taxi crash data from different geographical and institutional contexts to assess the transferability of the identified heterogeneous effects.
